# Supplementary material for: tauFisher predicts circadian time from a single sample of bulk and single-cell pseudobulk transcriptomic data
Source: Nat Commun. 2024 May 7;15:3840. doi: 10.1038/s41467-024-48041-6 (PMC11076472; doi:10.1038/s41467-024-48041-6)
Supplement: Supplementary file 1 — Supplementary Information [file 41467_2024_48041_MOESM1_ESM.pdf]

Supplementary Information

Benchmark Training

Since tauFisher relies on the `meta2d` function in the MetaCycle R package [1] to identify the periodic genes, if a training set does not include consecutive samples, we modified the input training data to have `NA`s for those samples. This may occur when the training set includes time points such as {4, 12, 16, ··· } and the "missing" value, 8, is assigned to the testing set. We then inserted a column for the "missing" value, 8, in the input training data for tauFisher and assigned `NA`s to all genes in that column. For the kidney and liver bulk RNAseq data sets [2], the `meta2d` function identified a large number (> 20) of significant genes with a period of 24 hours. To reduce the number of genes, we applied the following filters in order: (1) filtering out any genes that end with "Rik" or "-ps" or begin with "MT-" or "Gm", (2) having an amplitude greater than the mean amplitude, and (3) having an amplitude greater than the median amplitude.

Supplementary table 1: benchmark results with different accuracy definitions

Table 1 Benchmark results (mean ± standard deviation) when we used 80% of the data set for training and the remaining 20% for testing. Accuracy was calculated when different definitions of correctness were used. When a pipeline failed to predict for part of the 100 iterations, accuracy for the failed iteration is set to 0.

**Data**	**Metric**	**ZeitZeiger**	**TimeSignatR**	**tauFisher**	
			**w/ 2-sample**	**w/ Lomb-Scargle**	**w/ JTK_Cycle**
[2][2,4]	Accuracy (within 3 hr)	0.996 ± 0.028	**1.000 ± 0.000**	0.996 ± 0.028	**1.000 ± 0.000**
	Accuracy (within 2 hr)	0.986 ± 0.051	**1.000 ± 0.000**	0.966 ± 0.086	**1.000 ± 0.000**
	Accuracy (within 1 hr)	0.728 ± 0.179	0.904 ± 0.154	0.880 ± 0.158	**0.916 ± 0.128**
	Accuracy (exact)	0.004 ± 0.028	0.000 ± 0.000	**0.370 ± 0.219**	0.358 ± 0.209
[2][1,2,5]	Accuracy (within 3 hr)	NA	0.875 ± 0.279	0.720 ± 0.416	**0.945 ± 0.200**
	Accuracy (within 2 hr)	NA	0.620 ± 0.409	0.695 ± 0.414	**0.945 ± 0.200**
	Accuracy (within 1 hr)	NA	0.195 ± 0.333	0.565 ± 0.453	**0.815 ± 0.346**
	Accuracy (exact)	NA	0.000 ± 0.000	0.250 ± 0.314	**0.375 ± 0.344**
[2][3,4]	Accuracy (within 3 hr)	0.964 ± 0.100	**1.000 ± 0.000**	0.936 ± 0.133	0.986 ± 0.059
	Accuracy (within 2 hr)	0.868 ± 0.156	**0.988 ± 0.048**	0.880 ± 0.161	0.932 ± 0.103
	Accuracy (within 1 hr)	0.626 ± 0.225	0.844 ± 0.172	0.764 ± 0.196	**0.850 ± 0.162**
	Accuracy (exact)	0.002 ± 0.020	0.000 ± 0.000	**0.312 ± 0.189**	0.308 ± 0.219
[2][1,3,5]	Accuracy (within 3 hr)	NA	0.840 ± 0.340	0.840 ± 0.332	**0.970 ± 0.171**
	Accuracy (within 2 hr)	NA	0.565 ± 0.406	0.765 ± 0.344	**0.910 ± 0.269**
	Accuracy (within 1 hr)	NA	0.275 ± 0.385	0.585 ± 0.427	**0.735 ± 0.313**
	Accuracy (exact)	NA	0.000 ± 0.000	0.155 ± 0.263	**0.335 ± 0.326**
[2][6,4]	Accuracy (within 3 hr)	0.984 ± 0.061	**1.000 ± 0.000**	0.834 ± 0.166	0.922 ± 0.130
	Accuracy (within 2 hr)	0.924 ± 0.130	**0.998 ± 0.020**	0.790 ± 0.183	0.896 ± 0.141
	Accuracy (within 1 hr)	0.558 ± 0.219	**0.872 ± 0.152**	0.612 ± 0.218	0.682 ± 0.211
	Accuracy (exact)	0.002 ± 0.020	0.000 ± 0.000	0.240 ± 0.203	**0.242 ± 0.191**
[2][5,6]	Accuracy (within 3 hr)	NA	0.525 ± 0.457	0.420 ± 0.323	**0.595 ± 0.360**
	Accuracy (within 2 hr)	NA	0.280 ± 0.410	0.365 ± 0.347	**0.565 ± 0.353**
	Accuracy (within 1 hr)	NA	0.140 ± 0.257	0.300 ± 0.284	**0.410 ± 0.358**
	Accuracy (exact)	NA	0.000 ± 0.000	0.110 ± 0.208	**0.115 ± 0.211**
[2][4,7]	Accuracy (within 3 hr)	0.932 ± 0.121	**1.000 ± 0.000**	0.824 ± 0.183	0.904 ± 0.146
	Accuracy (within 2 hr)	0.786 ± 0.200	**0.990 ± 0.044**	0.760 ± 0.201	0.842 ± 0.166
	Accuracy (within 1 hr)	0.504 ± 0.226	**0.794 ± 0.192**	0.566 ± 0.222	0.608 ± 0.224
	Accuracy (exact)	0.004 ± 0.028	0.000 ± 0.000	**0.266 ± 0.182**	0.246 ± 0.203
[2][5,7]	Accuracy (within 3 hr)	NA	0.570 ± 0.444	0.360 ± 0.302	**0.810 ± 0.316**
	Accuracy (within 2 hr)	NA	0.305 ± 0.340	0.300 ± 0.293	**0.660 ± 0.382**
	Accuracy (within 1 hr)	NA	0.185 ± 0.272	0.185 ± 0.243	**0.430 ± 0.302**
	Accuracy (exact)	NA	0.000 ± 0.000	0.03 ± 0.119	**0.15 ± 0.241**
[3][4]	Accuracy (within 3 hr)	0.548 ± 0.105	**0.869 ± 0.095**	0.567 ± 0.132	0.563 ± 0.117
	Accuracy (within 2 hr)	0.400 ± 0.108	**0.720 ± 0.135**	0.414 ± 0.123	0.471 ± 0.126
	Accuracy (within 1 hr)	0.218 ± 0.097	**0.428 ± 0.142**	0.254 ± 0.108	0.299 ± 0.103
	Accuracy (exact)	0.006 ± 0.023	0.000 ± 0.000	0.086 ± 0.087	**0.103 ± 0.067**
[4][5]	Accuracy (within 3 hr)	0.601 ± 0.105	**0.914 ± 0.061**	0.407 ± 0.083	0.462 ± 0.114
	Accuracy (within 2 hr)	0.446 ± 0.111	**0.750 ± 0.095**	0.306 ± 0.074	0.370 ± 0.104
	Accuracy (within 1 hr)	0.213 ± 0.091	**0.452 ± 0.089**	0.197 ± 0.072	0.235 ± 0.082
	Accuracy (exact)	0.000 ± 0.000	0.000 ± 0.000	0.063 ± 0.033	**0.078 ± 0.045**
[5][4]	Accuracy (within 3 hr)	0.563 ± 0.350	**0.867 ± 0.171**	0.783 ± 0.248	0.750 ± 0.286
	Accuracy (within 2 hr)	0.460 ± 0.344	0.690 ± 0.269	**0.740 ± 0.258**	0.670 ± 0.330
	Accuracy (within 1 hr)	0.307 ± 0.275	0.427 ± 0.289	**0.590 ± 0.280**	0.543 ± 0.295
	Accuracy (exact)	0.010 ± 0.057	0.000 ± 0.000	**0.257 ± 0.263**	0.227 ± 0.241
[6][5]	Accuracy (within 3 hr)	0.108 ± 0.298	**0.932 ± 0.181**	0.610 ± 0.237	0.705 ± 0.237
	Accuracy (within 2 hr)	0.075 ± 0.218	**0.818 ± 0.251**	0.545 ± 0.239	0.635 ± 0.237
	Accuracy (within 1 hr)	0.040 ± 0.150	**0.542 ± 0.256**	0.412 ± 0.257	0.468 ± 0.253
	Accuracy (exact)	0.000 ± 0.000	0.000 ± 0.000	**0.190 ± 0.195**	0.182 ± 0.181

[1]If ZeitZeiger [7] was unable to do a 3-fold cross validation, we ran ZeitZeiger without any cross validation and set `sumabsv` = 1 and `nSpc` = 3.
[2] kidney [3] liver [4] microarray [5] bulk RNAseq [6] brainstem [7] cerebellum

Supplementary table 2: benchmark results

Table 2 Benchmark results (mean ± standard deviation) when we use 80% of the data set for training and the remaining 20% for testing. When a pipeline failed to predict for part of the 100 iterations, accuracy and RMSE for the failed iteration are set to 0 and 12 respectively.

Data	Metric	ZeitZeiger	TimeSignatR	tauFisher	
			w/ 2-sample	w/ Lomb-Scargle	w/ JTK_Cycle
[2][2,4]	Accuracy	0.986 ± 0.051	**1.000 ± 0.000**	0.966 ± 0.086	**1.000 ± 0.000**
	RMSE	0.849 ± 0.244	**0.568 ± 0.195**	1.021 ± 0.406	0.911 ± 0.256
	# NA	0	0	0	0
[2][1,2,5]	Accuracy	NA	0.620 ± 0.409	0.695 ± 0.414	**0.945 ± 0.200**
	RMSE	NA	2.035 ± 1.766	2.965 ± 3.117	**1.076 ± 0.981**
	# NA	100	0	0	0
[2][3,4]	Accuracy	0.868 ± 0.156	**0.988 ± 0.048**	0.880 ± 0.161	0.932 ± 0.103
	RMSE	1.391 ± 1.124	**0.689 ± 0.240**	1.797 ± 1.479	1.182 ± 0.451
	# NA	0	0	0	0
[2][1,3,5]	Accuracy	NA	0.565 ± 0.406	0.765 ± 0.344	**0.910 ± 0.269**
	RMSE	NA	2.262 ± 1.836	2.269 ± 2.356	**1.203 ± 0.825**
	# NA	100	0	0	0
[2][4,6]	Accuracy	0.924 ± 0.130	**0.998 ± 0.020**	0.790 ± 0.183	0.896 ± 0.141
	RMSE	1.148 ± 0.450	**0.633 ± 0.248**	2.603 ± 1.540	1.709 ± 0.994
	# NA	0	0	0	0
[2][5,6]	Accuracy	NA	0.280 ± 0.410	0.365 ± 0.347	**0.565 ± 0.353**
	RMSE	NA	3.586 ± 2.588	5.763 ± 2.645	**3.468 ± 1.882**
	# NA	100	0	0	0
[2][4,7]	Accuracy	0.786 ± 0.200	**0.990 ± 0.044**	0.760 ± 0.201	0.842 ± 0.166
	RMSE	1.526 ± 0.653	**0.751 ± 0.253**	2.683 ± 1.623	2.063 ± 1.262
	# NA	0	0	0	0
[2][5,7]	Accuracy	NA	0.305 ± 0.340	0.300 ± 0.293	**0.660 ± 0.382**
	RMSE	NA	3.255 ± 1.913	5.921 ± 2.656	**2.386 ± 1.405**
	# NA	100	0	0	0
[3][4]	Accuracy	0.400 ± 0.108	**0.720 ± 0.135**	0.414 ± 0.123	0.471 ± 0.126
	RMSE	4.609 ± 0.802	**2.598 ± 1.220**	4.998 ± 0.848	5.087 ± 0.845
	# NA	0	0	0	0
[4][5]	Accuracy	0.446 ± 0.111	**0.750 ± 0.095**	0.306 ± 0.074	0.370 ± 0.104
	RMSE	4.095 ± 0.806	**1.806 ± 0.367**	5.959 ± 0.547	5.481 ± 0.660
	# NA	0	0	1	1
[5][4]	Accuracy	0.46 ± 0.344	0.690 ± 0.269	**0.74 ± 0.258**	0.67 ± 0.330
	RMSE	4.558 ± 3.640	**1.822 ± 0.820**	2.488 ± 1.662	2.707 ± 1.976
	# NA	15	0	0	0
[6][5]	Accuracy	0.075 ± 0.218	**0.818 ± 0.251**	0.545 ± 0.239	0.635 ± 0.237
	RMSE	10.784 ± 3.317	**1.439 ± 1.101**	3.722 ± 1.880	2.969 ± 1.477
	# NA	88	0	0	0

[1]If ZeitZeiger [7] was unable to do a 3-fold cross validation, we ran ZeitZeiger without any cross validation and set `sumabsv` = 1 and `nSpc` = 3.

[2]kidney

[3]liver

[4]microarray

[5]bulk RNA

[6]brainstem

[7]cerebellum

Supplementary figures

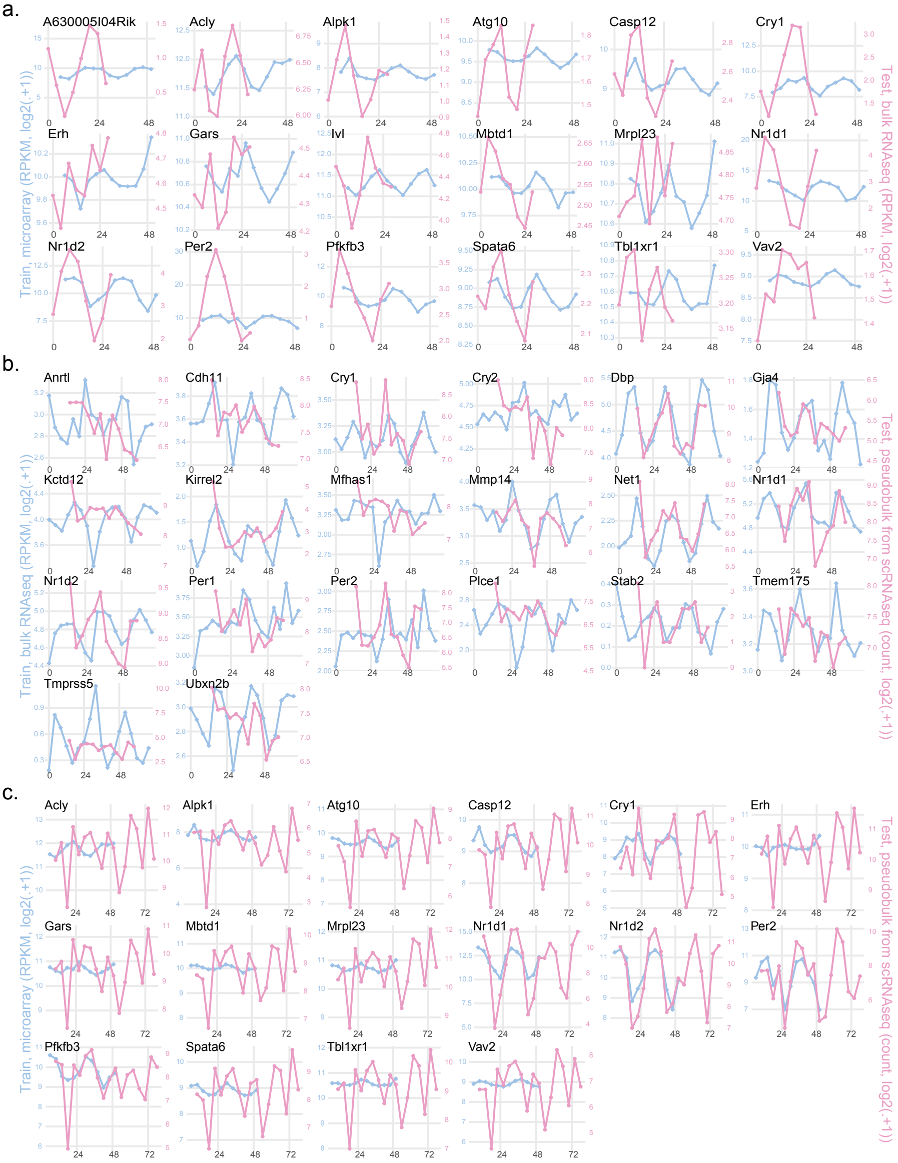

Fig. 1 Log2-transformed training and test data in the tauFisher pipeline. a Training: mouse skin microarray (GSE38622); test: mouse skin bulk RNAseq (GSE83855). **b** Training: mouse SCN bulk RNAseq (GSE157077); test: mouse SCN scRNAseq (GSE117295) pseudobulk. **c** Training: mouse skin microarray (GSE38622); test: mouse dermis scRNAseq (GSE223109) pseudobulk. Source data are provided as a Source Data file.

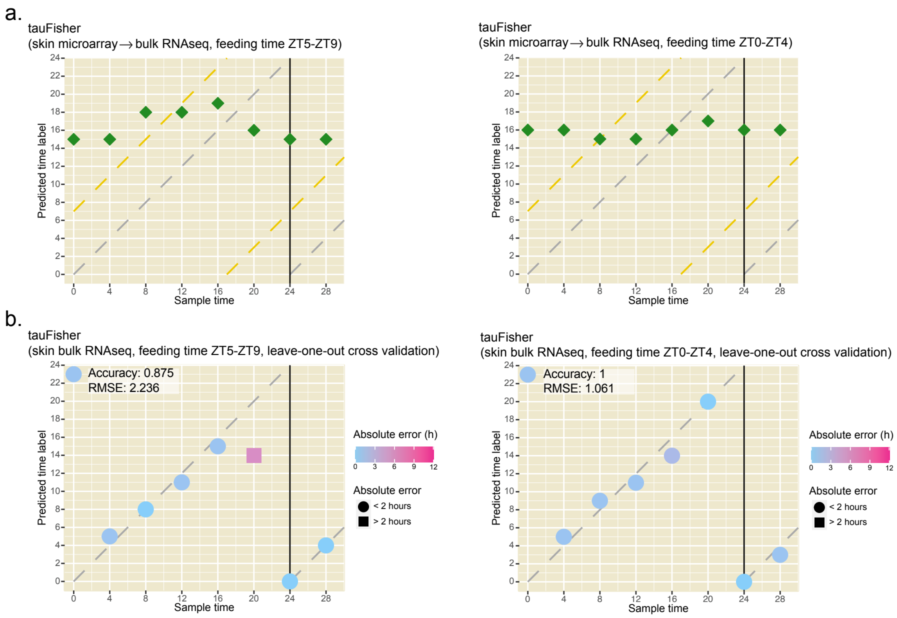

Fig. 2 tauFisher's performance in systems with disturbed circadian rhythms. **a** tauFisher trained on a control/healthy diurnal system predicts time labels that are away from the sample collection time of the tested disturbed systems. tauFisher was trained on skin microarray data collected from control/healthy mice (GSE38622) and tested on skin bulk RNAseq (GSE83855) collected from mice experienced time-restricted feeding schedules. Food was available between ZT5-ZT9 or ZT0-ZT4, while mice usually feed during early night (ZT12-ZT16). Grey dashed lines mark where predicted time labels equal sample collection time. Yellow dashed lines mark where predictions equal the internal circadian time if the internal circadian time is phase-shifted according to the feeding schedule, which is not true [8]. **b** tauFisher trained on data collected from a disturbed system can predict time labels of samples collected from the same system. Using skin bulk RNAseq (GSE83855) collected from mice experienced the two time-restricted feeding schedules mentioned above, we performed leave-one-out cross validation. Each sample in each of the two feeding schedules was reserved for testing while tauFisher trained on the remaining samples. Grey dashed lines mark where predicted time labels equal sample collection time. Source data are provided as a Source Data file.

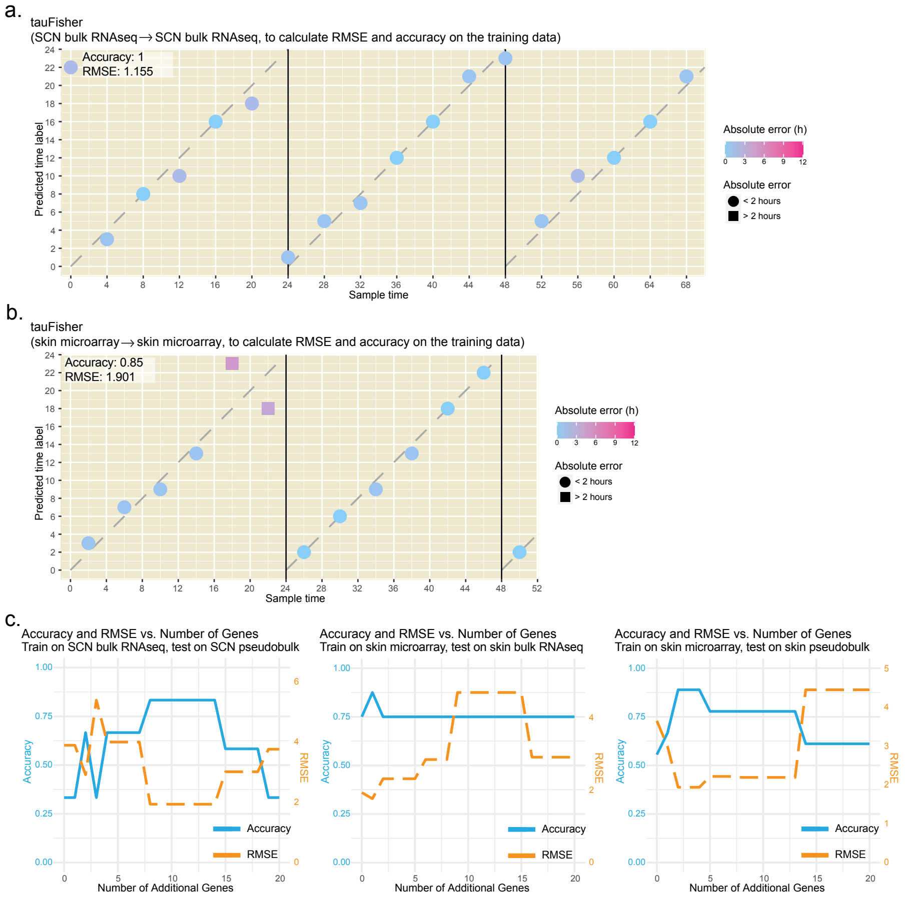

Fig. 3 tauFisher's performance within the training data and its sensitivity to the number of genes used in the pipeline. a Prediction results when tauFisher was trained on mouse SCN bulk RNAseq (GSE157077) and tested on the same data. **b** Prediction results when tauFisher was trained on mouse skin microarray (GSE38622) and tested on the same data. **a-b** Grey dashed lines mark where predictions equal sample collection time. **c** tauFisher's performance is sensitive to the number of additional predictor genes used in the pipeline. We performed a sensitivity analysis and calculated accuracy and RMSE when we chose different numbers of diurnal genes in addition to the core clock genes with 24-hour period length. Although including 10 additional genes does not guarantee the best performance, it is a reasonable choice across different datasets. Source data are provided as a Source Data file.

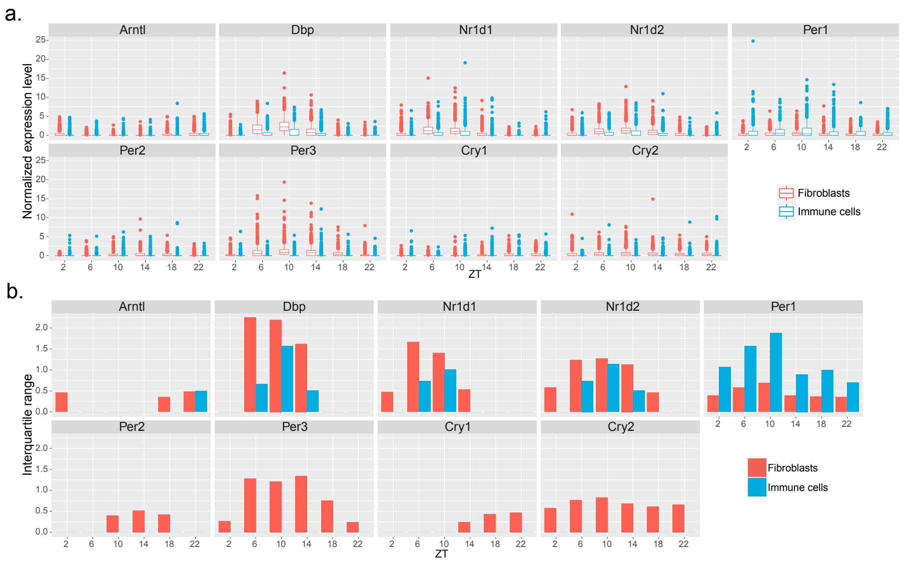

Fig. 4 Expression ranges of the core clock genes are similar in dermal fibroblasts and immune cells. a Box plots of expression levels of core clock genes in fibroblasts (red) and immune cells (blue). Expression value is normalized as transcript counts divided by total reads in each cell times 10,000. The ranges of the expression levels of the core clock genes are similar in fibroblasts and immune cells. The line inside each box indicates the median. Bounds of box represent the first and third quartiles. The upper and lower whiskers extend to the largest and smallest value within 1.5 times the inter-quartile range respectively. **b** Interquartile range of the expression of core clock genes in fibroblasts (red) and immune cells (blue). The measurements in fibroblasts are more variable than the measurements in immune cells in general. n = 3 biologically independent samples per circadian time point. Source data are provided as a Source Data file.

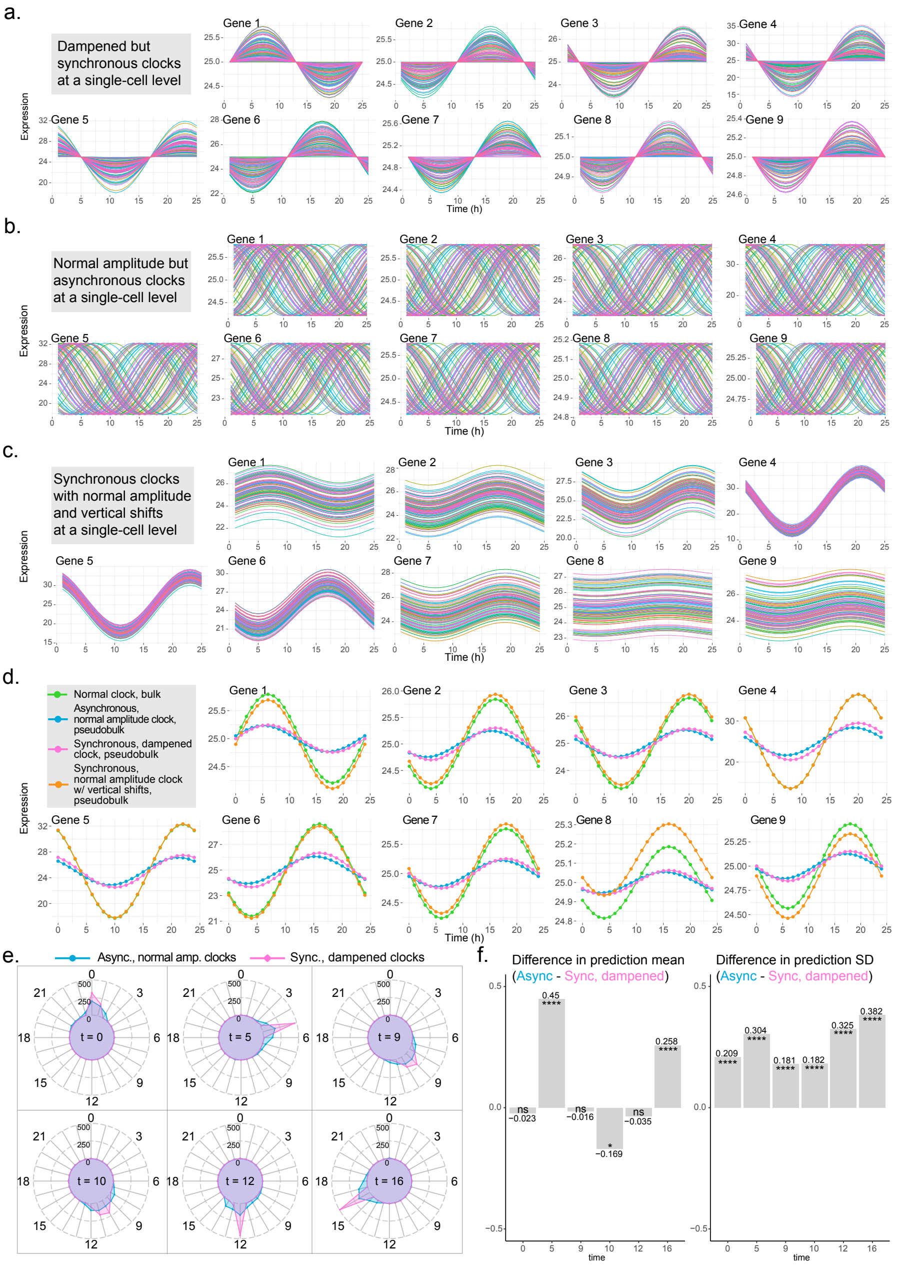

Fig. 5 Legend on the next page.

Fig. 5 tauFisher's ability to determine circadian phase heterogeneity in simulated data. **a** Simulated expression of nine diurnal genes in 100 single cells that have synchronous circadian clocks with dampened amplitudes. **b** Simulated expression of nine diurnal genes in 100 single cells that have asynchronous circadian clocks but normal amplitudes. **c** Simulated expression of nine diurnal genes in 100 single cells that have synchronous circadian clocks with normal amplitudes but different mean expression level over time (vertical shifts). Because we needed to increase ranges of y-axis to plot all the curves, some curves may appear to be dampened (i.g. Gene 8). **a-c** Each line represents gene expression in a cell over time. **d** At the bulk level, scenarios in **a** and **b** generate similar patterns, which are oscillations with dampened amplitudes but similar peaking time when compared to a normal bulk-level clock. Scenario in **c** does not produce dampened amplitudes at the pseudobulk level. **e** We combined tauFisher with bootstrapping and randomly selected six time points to do the comparison. We generated 500 predictions and plotted the distributions for the cells in **a** (pink) and **b**(blue). **f** Bar plots showing the differences between the prediction mean (left) and standard deviation (right) at each time point (cells in **b** - cells in **a**). ns: p-value > 0.05, $*$: p-value ≤ 0.05, $****$: p-value ≤ 0.0001. P-values are determined using Rao's Tests for Homogeneity and the Wallraff Test of Angular Distances. Source data are provided as a Source Data file.

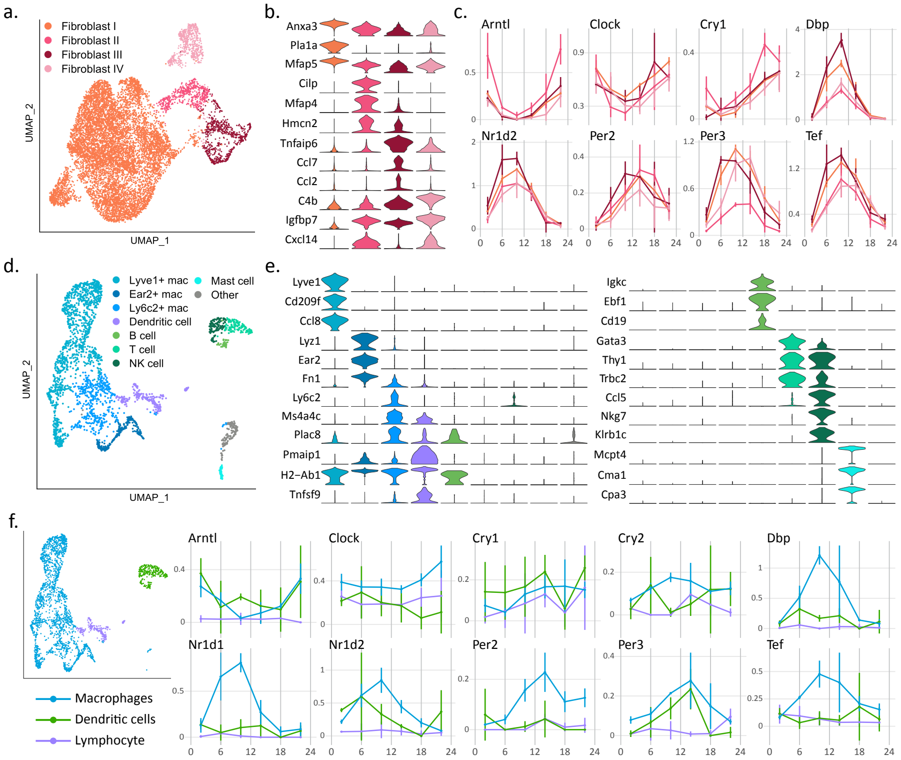

Fig. 6 Subclustering of mouse dermal fibroblasts and immune cells. **a** Four subclusters were identified for the dermal fibroblasts. **b** Violin plots show expression of marker genes for each fibroblast subcluster. **c** Core clock gene expression for the four fibroblast subclusters show similar rhythmic pattern. **d** Eight immune subclusters including both myeloid cells and lymphoid cells were identified. **e** Violin plots show expression of marker genes for each immune subcluster. **f** Due to low cell counts for some time points, we combined the eight subclusters of immune cells to form three major clusters. The core clock gene expression over time is plotted for the three major clusters. With the great variability present in the data, probably contributed by low cell counts, the core clock genes do not show robust rhythms. **c, f** n = 3 biologically independent samples per circadian time point. Data are presented as mean values +/- SD. Source data are provided as a Source Data file.

Supplementary References

[1] Wu, G., Anafi, R.C., Hughes, M.E., Kornacker, K., Hogenesch, J.B.: Metacycle: an integrated r package to evaluate periodicity in large scale data. Bioinformatics **32**, 3351–3353 (2016). https://doi.org/10.1093/BIOINFORMATICS/BTW405

[2] Zhang, R., Lahens, N.F., Ballance, H.I., Hughes, M.E., Hogenesch, J.B.: A circadian gene expression atlas in mammals: Implications for biology and medicine. Proceedings of the National Academy of Sciences **111**, 16219–16224 (2014). https://doi.org/10.1073/PNAS.1408886111

[3] Arnardottir, E.S., Nikonova, E.V., Shockley, K.R., Podtelezhnikov, A.A., Anafi, R.C., Tanis, K.Q., Maislin, G., Stone, D.J., Renger, J.J., Winrow, C.J., Pack, A.I.: Blood-gene expression reveals reduced circadian rhythmicity in individuals resistant to sleep deprivation. Sleep **37**, 1589–1600 (2014). https://doi.org/10.5665/SLEEP.4064

[4] Braun, R., Kath, W.L., Iwanaszko, M., Kula-Eversole, E., Abbott, S.M., Reid, K.J., Zee, P.C., Allada, R.: Universal method for robust detection of circadian state from gene expression. Proceedings of the National Academy of Sciences **115**, 9247–9256 (2018). https://doi.org/10.1073/PNAS.1800314115

[5] Geyfman, M., Kumar, V., Liu, Q., Ruiz, R., Gordon, W., Espitia, F., Cam, E., Millar, S.E., Smyth, P., Ihler, A., Takahashi, J.S., Andersen, B.: Brain and muscle arnt-like protein-1 (bmal1) controls circadian cell proliferation and susceptibility to uvb-induced dna damage in the epidermis. Proceedings of the National Academy of Sciences of the United States of America **109**, 11758 (2012). https://doi.org/10.1073/PNAS.1209592109

[6] Tognini, P., Samad, M., Kinouchi, K., Liu, Y., Helbling, J.-C., Moisan, M.-P., Eckel-Mahan, K.L., Baldi, P., Sassone-Corsi, P.: Reshaping circadian metabolism in the suprachiasmatic nucleus and prefrontal cortex by nutritional challenge. Proceedings of the National Academy of Sciences **117**, 29904–29913 (2020). https://doi.org/10.1073/PNAS.2016589117

[7] Hughey, J.J., Hastie, T., Butte, A.J.: Zeitzeiger: Supervised learning for high-dimensional data from an oscillatory system. Nucleic Acids Research **44**, 80 (2016). https://doi.org/10.1093/nar/gkw030

[8] Wang, H., Spyk, E.V., Liu, Q., Geyfman, M., Salmans, M., Kumar, V., Ihler, A., Li, N., Takahashi, J.S., Andersen, B.: Time-restricted feeding shifts the skin circadian clock and alters uvb-induced dna damage. Cell Reports **20**, 1061–1072 (2017). https://doi.org/10.1016/j.celrep.2017.07.022
