# Supplementary material for: tauFisher predicts circadian time from a single sample of bulk and single-cell pseudobulk transcriptomic data
Source: Nat Commun. 2024 May 7;15:3840. doi: 10.1038/s41467-024-48041-6 (PMC11076472; doi:10.1038/s41467-024-48041-6)
Supplement: Supplementary file 3 — Description of Additional Supplementary Files [file 41467_2024_48041_MOESM3_ESM.pdf]

Description of Additional Supplementary Files:

Supplementary Data 1: Output from meta2d, using scRNAseq pseudobulk data calculated from dermal fibroblasts as the input. There are 1946 significantly rhythmic genes (JTK_p-value < 0.05) in dermal fibroblasts.

Supplementary Data 2: Output from meta2d, using scRNAseq pseudobulk data calculated from dermal immune cells as the input. There are 432 significantly rhythmic genes (JTK_p-value < 0.05) in dermal immune cells.

Supplementary Data 3: Gene Ontology terms from clusterProfiler applied to different quarter-day rhythmic genes lists from dermal fibroblasts (Methods). Quarter-day information is stored in the quarter-day column (morning, day, evening, night). Terms with p-value < 0.05 were considered significant in the manuscript.

Supplementary Data 4: Gene Ontology terms from clusterProfiler applied to different quarter-day rhythmic genes lists from dermal immune cells (Methods). Quarter-day information is stored in the quarter-day column (morning, day, evening, night). Terms with p-value < 0.05 were considered significant in the manuscript.

Supplementary Data 5: Within-sample normalized training data: tauFisher was trained on mouse skin microarray data (GSE38622) and tested on mouse skin bulk RNAseq data (GSE83855). Columns, gene pairs; rows, time points.

Supplementary Data 6: Within-sample normalized test data: tauFisher was trained on mouse skin microarray data (GSE38622) and tested on mouse skin bulk RNAseq data (GSE83855). Columns, gene pairs; rows, samples.
