# Supplementary material for: tauFisher predicts circadian time from a single sample of bulk and single-cell pseudobulk transcriptomic data
Source: Nat Commun. 2024 May 7;15:3840. doi: 10.1038/s41467-024-48041-6 (PMC11076472; doi:10.1038/s41467-024-48041-6)
Supplement: Supplementary file 10 — Reporting Summary [file 41467_2024_48041_MOESM10_ESM.pdf]

nature portfolio

Corresponding author(s): Bogi Andersen, John Lowengrub, Babak Shahbaba

Last updated by author(s): Mar 8, 2024

Reporting Summary

Nature Portfolio wishes to improve the reproducibility of the work that we publish. This form provides structure for consistency and transparency in reporting. For further information on Nature Portfolio policies, see our Editorial Policies and the Editorial Policy Checklist.

Statistics

For all statistical analyses, confirm that the following items are present in the figure legend, table legend, main text, or Methods section.

n/a	Confirmed	
☐	☒	The exact sample size (*n*) for each experimental group/condition, given as a discrete number and unit of measurement
☐	☒	A statement on whether measurements were taken from distinct samples or whether the same sample was measured repeatedly
☐	☒	The statistical test(s) used AND whether they are one- or two-sided<br>*Only common tests should be described solely by name; describe more complex techniques in the Methods section.*
☒	☐	A description of all covariates tested
☐	☒	A description of any assumptions or corrections, such as tests of normality and adjustment for multiple comparisons
☐	☒	A full description of the statistical parameters including central tendency (e.g. means) or other basic estimates (e.g. regression coefficient) AND variation (e.g. standard deviation) or associated estimates of uncertainty (e.g. confidence intervals)
☐	☒	For null hypothesis testing, the test statistic (e.g. *F*, *t*, *r*) with confidence intervals, effect sizes, degrees of freedom and *P* value noted<br>*Give P values as exact values whenever suitable.*
☒	☐	For Bayesian analysis, information on the choice of priors and Markov chain Monte Carlo settings
☒	☐	For hierarchical and complex designs, identification of the appropriate level for tests and full reporting of outcomes
☒	☐	Estimates of effect sizes (e.g. Cohen's *d*, Pearson's *r*), indicating how they were calculated

Our web collection on statistics for biologists contains articles on many of the points above.

Software and code

Policy information about availability of computer code

Data collection	We used Cell Ranger version 3.1.0 with MM10 reference to process the raw sequencing output.
Data analysis	tauFisher at https://github.com/micnngo/tauFisher; TimeSignatR at https://github.com/braunr/TimeSignatR; ZeitZeiger at https://github.com/hugheylab/zeitzeiger.<br>Seurat V3; ClusterProfiler (4.2.2); circular (0.5.0); MetaCycle (1.2.0); scales (1.3.0); oligo (1.54.1); biomaRt (2.46.3); clusterProfiler (4.4.0); CellRanger (3.1.0)

For manuscripts utilizing custom algorithms or software that are central to the research but not yet described in published literature, software must be made available to editors and reviewers. We strongly encourage code deposition in a community repository (e.g. GitHub). See the Nature Portfolio guidelines for submitting code & software for further information.

Data

Policy information about availability of data

All manuscripts must include a data availability statement. This statement should provide the following information, where applicable:

- Accession codes, unique identifiers, or web links for publicly available datasets
- A description of any restrictions on data availability
- For clinical datasets or third party data, please ensure that the statement adheres to our policy

All published datasets used in this paper can be accessed through their respective GEO accession codes. The time series of microarray data collected from mouse

kidney, liver, brainstem and cerebellum are available under GSE54650. The time series of bulk RNAseq data collected from mouse kidney, liver, brainstem and cerebellum are available under GSE54651. The time series of microarray data collected from mouse skin are available under GSE38622. The time series of bulk RNAseq data from mouse SCN are available under GSE157077. The time series of scRNAseq data from mouse SCN are available under GSE117295. The bulk RNAseq data collected from mouse skin in control and time-restricted feeding conditions are available under GSE83855. Although the two human blood datasets are accessible through their GEO accession codes GSE56931 and GSE113883, this paper used the versions provided in the TimeSignatR package: https://github.com/braunr/TimeSignatR.
The time series of scRNAseq data from mouse dermal skin collected in this study are available in the GEO database under GSE223109.

Research involving human participants, their data, or biological material

Policy information about studies with human participants or human data. See also policy information about sex, gender (identity/presentation), and sexual orientation and race, ethnicity and racism.

Reporting on sex and gender	N/A
Reporting on race, ethnicity, or other socially relevant groupings	N/A
Population characteristics	N/A
Recruitment	N/A
Ethics oversight	N/A

Note that full information on the approval of the study protocol must also be provided in the manuscript.

Field-specific reporting

Please select the one below that is the best fit for your research. If you are not sure, read the appropriate sections before making your selection.

☒ Life sciences ☐ Behavioural & social sciences ☐ Ecological, evolutionary & environmental sciences

For a reference copy of the document with all sections, see nature.com/documents/nr-reporting-summary-flat.pdf

Life sciences study design

All studies must disclose on these points even when the disclosure is negative.

Sample size	Mouse dorsal dermal skin samples were collected every 4 hours in a 24-hour day for three days to generate 18 scRNAseq data from 18 mice in total. For each circadian time point: n = 3. Total sample size: n = 18. No statistical method was used to predetermine sample size. We chose to collect three biological replicates per circadian time point because previous circadian gene expression experiments showed that n = 3 allowed robust detection of circadian genes
Data exclusions	No dataset was excluded from the experiment.
Replication	All attempts at replication were successful and there are three biological replicates at each circadian time point.
Randomization	There was no experimental group involved. Mice were maintained under the same condition with the regular 12-12 light-dark cycle schedule. We randomly obtained a mouse at the time of sample collection.
Blinding	During the collection and analysis of the scRNAseq data collected from mouse dermal skin in this study, the investigators were not blinded to time labels.

Reporting for specific materials, systems and methods

We require information from authors about some types of materials, experimental systems and methods used in many studies. Here, indicate whether each material, system or method listed is relevant to your study. If you are not sure if a list item applies to your research, read the appropriate section before selecting a response.

Materials & experimental systems

n/a	Involved in the study
x	Antibodies
x	Eukaryotic cell lines
x	Palaeontology and archaeology
	x Animals and other organisms
x	Clinical data
x	Dual use research of concern
x	Plants

Methods

n/a	Involved in the study
x	ChIP-seq
x	Flow cytometry
x	MRI-based neuroimaging

Animals and other research organisms

Policy information about studies involving animals; ARRIVE guidelines recommended for reporting animal research, and Sex and Gender in Research

Laboratory animals	The animals used in the study were wild type adult male C57BL/6 mice. They were kept under the regular 12-12 light-dark cycle with ambient temperature around 71F and humidity at approximately 45%. They were eight weeks old (postnatal day ~54) at the time of sample collection.
Wild animals	The study did not involve wild animals.
Reporting on sex	Only male mice were used
Field-collected samples	The study did not use field-collected samples.
Ethics oversight	Institutional Animal Care and Use Committee (IACUC) at University of California Irvine

Note that full information on the approval of the study protocol must also be provided in the manuscript.

Plants

Seed stocks	N/A
Novel plant genotypes	N/A
Authentication	N/A
